# Supplementary material for: Cascading epigenomic analysis for identifying disease genes from the regulatory landscape of GWAS variants
Source: PLoS Genet. 2021 Nov 22;17(11):e1009918. doi: 10.1371/journal.pgen.1009918 (PMC8648125; doi:10.1371/journal.pgen.1009918)
Supplement: S1 Fig — Ratio of the number of significant PCs extracted from the significant genes over the number of significant PCs extracted from the tested genes displayed. The number of significant PCs was used as an estimate of the number of distinct signals within a set of genes (see Methods). This analysis accounts for how some SNPs are shared between models of spatially proximal genes. The overall trend of CEWAS attaining higher detection rate than MetaXcan and EpiXcan remains with this analysis. (PDF) [file pgen.1009918.s007.pdf]

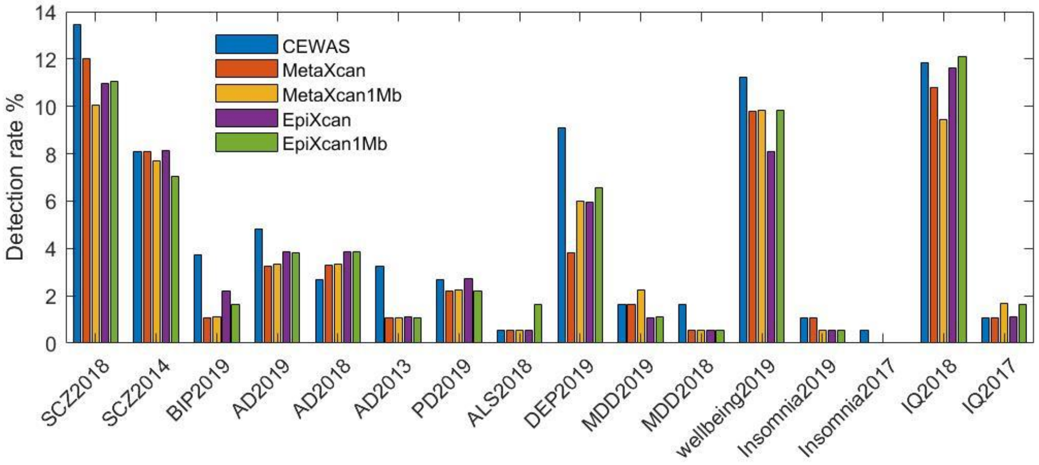

S1 Fig. Detection rate based on distinct signals. Ratio of the number of significant PCs extracted from the significant genes over the number of significant PCs extracted from the tested genes displayed. The number of significant PCs was used as an estimate of the number of distinct signals within a set of genes (see Methods). This analysis accounts for how some SNPs are shared between models of spatially proximal genes. The overall trend of CEWAS attaining higher detection rate than MetaXcan and EpiXcan remains with this analysis.
