# Supplementary material for: Cascading epigenomic analysis for identifying disease genes from the regulatory landscape of GWAS variants
Source: PLoS Genet. 2021 Nov 22;17(11):e1009918. doi: 10.1371/journal.pgen.1009918 (PMC8648125; doi:10.1371/journal.pgen.1009918)
Supplement: S2 Fig — (a) R2 estimated by correlating the predicted and measured expression levels in ROSMAP. CEWAS attained lower R2 as expected, since CEWAS is designed to extract a specific component of gene expression, namely the epigenomic component that is driven by genetic effects. In contrast, MetaXcan and EpiXcan are optimized for predicting gene expression. (b) R2 estimated by applying ROSMAP models to CMC genotype data and correlating the predicted expression levels with that measured in CMC. R2 appears similar across methods, but CEWAS actually attained slightly lower R2 if we zoom into the results (see S3 Fig). (PDF) [file pgen.1009918.s008.pdf]

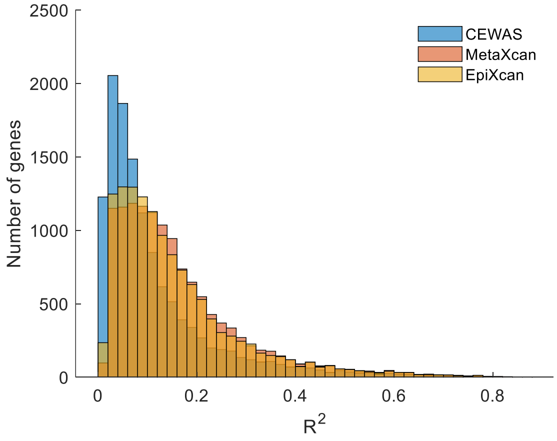

(a) Prediction on ROSMAP data

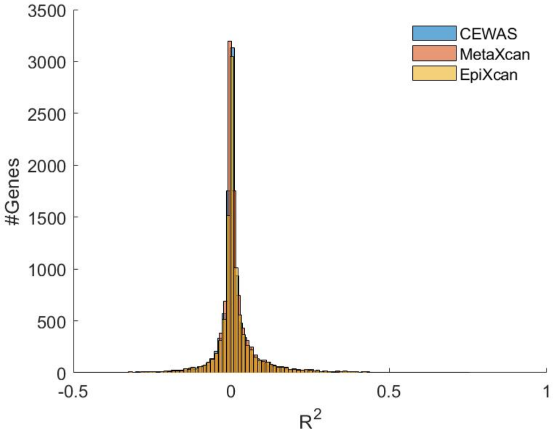

(b) Prediction on CMC data

S2 Fig. R^2 of gene models. (a) R^2 estimated by correlating the predicted and measured expression levels in ROSMAP. CEWAS attained lower R^2 as expected, since CEWAS is designed to extract a specific component of gene expression, namely the epigenomic component that is driven by genetic effects. In contrast, MetaXcan and EpiXcan are optimized for predicting gene expression. (b) R^2 estimated by applying ROSMAP models to CMC genotype data and correlating the predicted expression levels with that measured in CMC. R^2 appears similar across methods, but CEWAS actually attained slightly lower R^2 if we zoom into the results (see Fig S3).
