# Supplementary material for: Cascading epigenomic analysis for identifying disease genes from the regulatory landscape of GWAS variants
Source: PLoS Genet. 2021 Nov 22;17(11):e1009918. doi: 10.1371/journal.pgen.1009918 (PMC8648125; doi:10.1371/journal.pgen.1009918)
Supplement: S3 Fig — Models trained with ROSMAP data were applied to CMC genotype data to predict gene expression. Correlation attained by CEWAS is slightly lower than MetaXcan and EpiXcan. (PDF) [file pgen.1009918.s009.pdf]

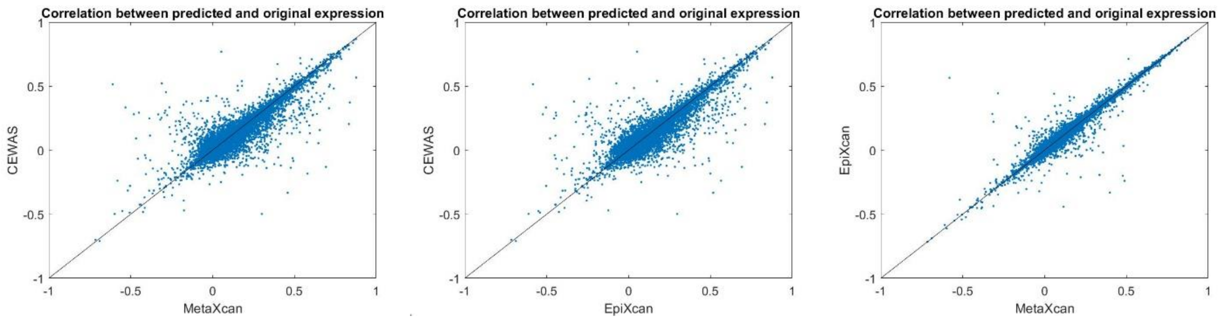

S3 Fig. Correlation between predicted and measured expression levels on CMC data. Models trained with ROSMAP data were applied to CMC genotype data to predict gene expression. Correlation attained by CEWAS is slightly lower than MetaXcan and EpiXcan.
