# Supplementary material for: Cascading epigenomic analysis for identifying disease genes from the regulatory landscape of GWAS variants
Source: PLoS Genet. 2021 Nov 22;17(11):e1009918. doi: 10.1371/journal.pgen.1009918 (PMC8648125; doi:10.1371/journal.pgen.1009918)
Supplement: S4 Fig — Bars show the log p-value of enrichment of mQTLs and eQTLs assessed by LDSC for each GWAS. (a) The enrichment for mQTLs remains higher than for eQTLs with the window size of eQTLs matched to that of mQTLs (p = 0.0084, Wilcoxon sign rank test across GWAS). (b) The same trend holds but to a lesser extent when we matched the number of mQTL SNPs to the number of eQTL SNPs by taking mQTL SNPs that are closer to CpGs (p = 0.1122, Wilcoxon sign rank test across GWAS; p = 0.0269, paired t-test). (PDF) [file pgen.1009918.s010.pdf]

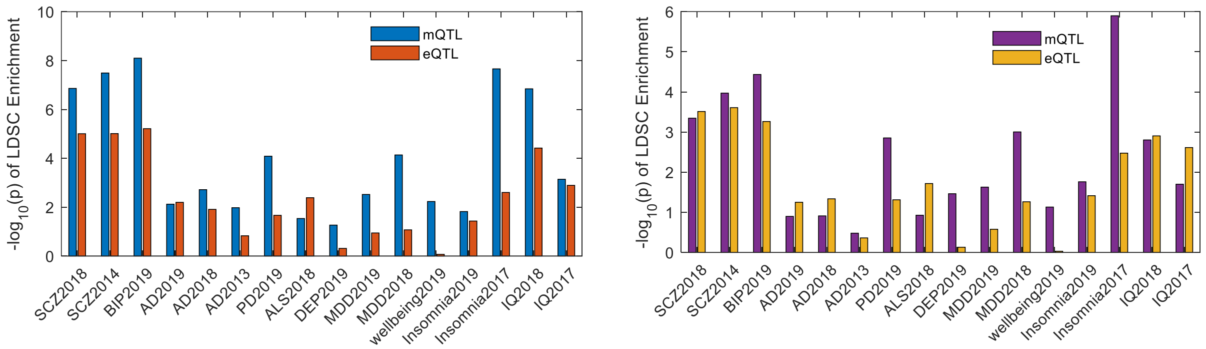

(a) mQTL window = eQTL window = 100Kb

(b) mQTL window = 100Kb, eQTL window = 1Mb

S4 Fig. Stability analysis of partitioned heritability. Bars show the log p-value of enrichment of mQTLs and eQTLs assessed by LDSC for each GWAS. (a) The enrichment for mQTLs remains higher than for eQTLs with the window size of eQTLs matched to that of mQTLs ($p=0.0084$, Wilcoxon sign rank test across GWAS). (b) The same trend holds but to a lesser extent when we matched the number of mQTL SNPs to the number of eQTL SNPs by taking mQTL SNPs that are closer to CpGs ($p=0.1122$, Wilcoxon sign rank test across GWAS; $p=0.0269$, paired t-test).
