# Supplementary material for: Cascading epigenomic analysis for identifying disease genes from the regulatory landscape of GWAS variants
Source: PLoS Genet. 2021 Nov 22;17(11):e1009918. doi: 10.1371/journal.pgen.1009918 (PMC8648125; doi:10.1371/journal.pgen.1009918)
Supplement: S5 Fig — Using mQTLs in Jaffe et al., [42] as the reference, we first found the top mQTL SNP for each CpG, and ordered the resulting top mQTLs based on p-values. Taking q% of the top mQTLs as “ground truth”, q = 5% to 50%, we computed AUC on mQTL p-values derived from the ROSMAP data. Since mQTLs from Jaffe et al. were estimated with a 20Kb window, we restricted the ROSMAP mQTLs to those within the same window. The same procedure was applied to estimate AUC for eQTLs, with eQTLs from CMC as the reference. Since eQTLs from CMC were estimated with a 1Mb window, we first examined ROSMAP eQTLs within a 1Mb window, but also examined CMC and ROSMAP eQTLs restricted to the same window size as the mQTLs, i.e. 20Kb. To estimate the null, we permuted the top eQTLs/mQTLs and repeated the procedure. (PDF) [file pgen.1009918.s011.pdf]

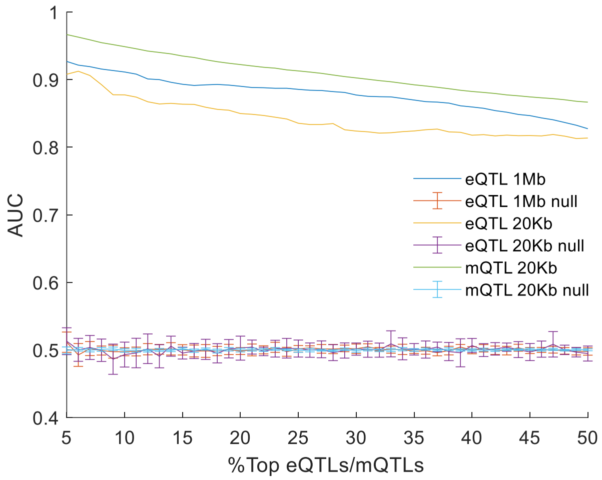

S5 Fig. Replication of mQTLs vs. eQTLs. Using mQTLs in Jaffe et al.[35] as the reference, we first found the top mQTL SNP for each CpG, and ordered the resulting top mQTLs based on p-values. Taking *q*% of the top mQTLs as "ground truth", *q* = 5% to 50%, we computed AUC on mQTL p-values derived from the ROSMAP data. Since mQTLs from Jaffe et al. were estimated with a 20Kb window, we restricted the ROSMAP mQTLs to those within the same window. The same procedure was applied to estimate AUC for eQTLs, with eQTLs from CMC as the reference. Since eQTLs from CMC were estimated with a 1Mb window, we first examined ROSMAP eQTLs within a 1Mb window, but also examined CMC and ROSMAP eQTLs restricted to the same window size as the mQTLs, i.e. 20Kb. To estimate the null, we permuted the top eQTLs/mQTLs and repeated the procedure.
