# Supplementary material for: Cascading epigenomic analysis for identifying disease genes from the regulatory landscape of GWAS variants
Source: PLoS Genet. 2021 Nov 22;17(11):e1009918. doi: 10.1371/journal.pgen.1009918 (PMC8648125; doi:10.1371/journal.pgen.1009918)
Supplement: S6 Fig — We assessed DNAm mediated effects on GWAS phenotypes in four ways. First, we examined results from models used in the first stage of CEWAS, i.e. MetaXcan models built with each CpG taken as the response and SNPs within ±50Kb from that CpG as predictors. Detection rate of these DNAm MetaXcan models, defined as the number of significant CpGs among tested CpGs, is significantly lower than CEWAS (p = 0.0004), MetaXcan (p = 0.0013), and EpiXcan (p = 0.0006) based on Wilcoxon sign rank test across GWAS. Second, we assessed CpG-to-gene mapping that does not use expression data, by taking the DNAm MetaXcan p-value of the closest CpG of each gene as the p-value of that gene. The gene level detection rate is significantly lower than CEWAS (p = 0.0004), MetaXcan (p = 0.0003), and EpiXcan (p = 0.0004). Third, we took the DNAm MetaXcan p-value of the CpG with the largest R2 in terms of DNAm prediction among CpGs within ±500Kb from each gene as the p-value of that gene. The detection rate is lower than CEWAS (p = 0.1331) and EpiXcan (p = 0.3808), and higher than MetaXcan (p = 0.4235). Fourth, to contrast using epigenomic annotation against explicitly modeling associations between SNPs and CpGs, we modified EpiXcan by weighting the sparse penalty using mQTL p-values. We first associated each CpG to SNPs that are within ±50Kb. We then found the smallest p-value, pk, across CpGs for each SNP k, and used 1010pk+0.5 (capped at 1) as the weight for its sparse penalty. Multiplying by 1010 accounts for the number of SNP-CpG pairs tested, and +0.5 penalizes SNPs with the strongest mQTL effects by half the amount as SNPs with weak/no mQTL effects. The detection rate of mQTL-weighted EpiXcan is significantly higher than both MetaXcan (p = 0.00006) and EpiXcan (p = 0.00006), and lower than CEWAS on average (p = 0.3881). (PDF) [file pgen.1009918.s012.pdf]

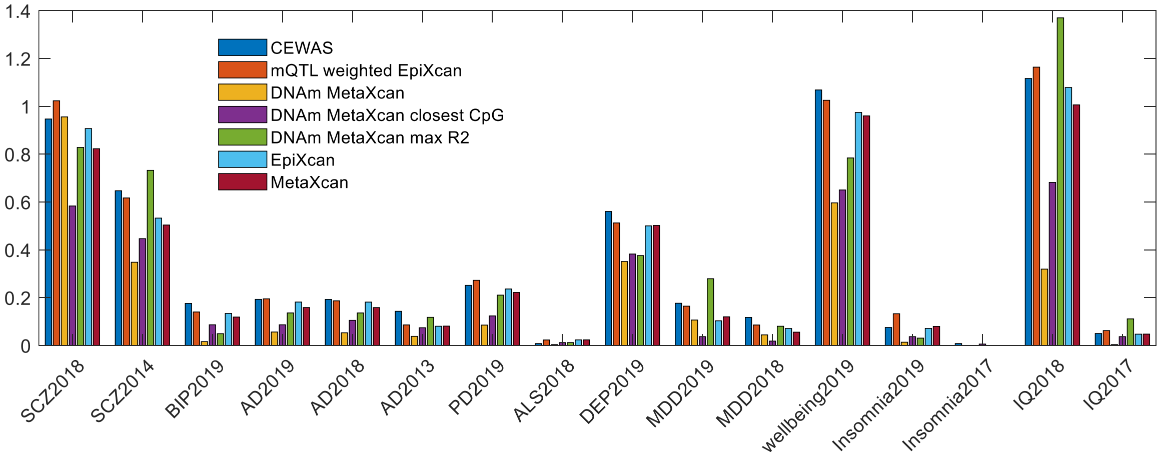


**S6 Fig. Gene detection rate with DNAm mediated effects on GWAS phenotypes.** We assessed DNAm mediated effects on GWAS phenotypes in four ways. First, we examined results from models used in the first stage of CEWAS, i.e. MetaXcan models built with each CpG taken as the response and SNPs within ±50Kb from that CpG as predictors. Detection rate of these DNAm MetaXcan models, defined as the number of significant CpGs among tested CpGs, is significantly lower than CEWAS (p=0.0004), MetaXcan (p=0.0013), and EpiXcan (p=0.0006) based on Wilcoxon sign rank test across GWAS. Second, we assessed CpG-to-gene mapping that does not use expression data, by taking the DNAm MetaXcan p-value of the closest CpG of each gene as the p-value of that gene. The gene level detection rate is significantly lower than CEWAS (p=0.0004), MetaXcan (p=0.0003), and EpiXcan (p=0.0004). Third, we took the DNAm MetaXcan p-value of the CpG with the largest $R^2$ in terms of DNAm prediction among CpGs within ±500Kb from each gene as the p-value of that gene. The detection rate is lower than CEWAS (p=0.1331) and EpiXcan (p=0.3808), and higher than MetaXcan (p=0.4235). Fourth, to contrast using epigenomic annotation against explicitly modeling associations between SNPs and CpGs, we modified EpiXcan by weighting the sparse penalty using mQTL p-values. We first associated each CpG to SNPs that are within ±50Kb. We then found the smallest p-value, $p_k$, across CpGs for each SNP $k$, and used $10^{10}p_k$+0.5 (capped at 1) as the weight for its sparse penalty. Multiplying by $10^{10}$ accounts for the number of SNP-CpG pairs tested, and +0.5 penalizes SNPs with the strongest mQTL effects by half the amount as SNPs with weak/no mQTL effects. The detection rate of mQTL-weighted EpiXcan is significantly higher than both MetaXcan (p=0.00006) and EpiXcan (p=0.00006), and lower than CEWAS on average (p=0.3881).
