# Supplementary material for: Cascading epigenomic analysis for identifying disease genes from the regulatory landscape of GWAS variants
Source: PLoS Genet. 2021 Nov 22;17(11):e1009918. doi: 10.1371/journal.pgen.1009918 (PMC8648125; doi:10.1371/journal.pgen.1009918)
Supplement: S7 Fig — Gene level z-scores estimated by CEWAS for schizophrenia vs. IQ displayed. A genomic correlation of -0.15 was observed. The genes that were detected by CEWAS across schizophrenia, bipolar disorder, depression, and IQ are highlighted in bold. Observing opposite signs in z-scores for the highlighted genes matches how risk alleles in these loci were shown to correlate with lower cognitive test scores [45]. (PDF) [file pgen.1009918.s013.pdf]

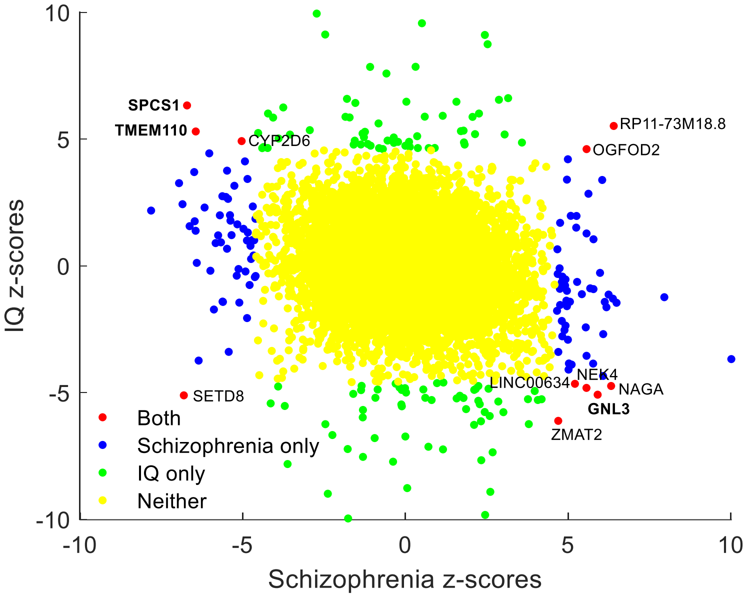

S7 Fig. Genomic correlation between schizophrenia and IQ. Gene level z-scores estimated by CEWAS for schizophrenia vs. IQ displayed. A genomic correlation of -0.15 was observed. The genes that were detected by CEWAS across schizophrenia, bipolar disorder, depression, and IQ are highlighted in bold. Observing opposite signs in z-scores for the highlighted genes matches how risk alleles in these loci were shown to correlate with lower cognitive test scores[37].
