# Supplementary material for: Cascading epigenomic analysis for identifying disease genes from the regulatory landscape of GWAS variants
Source: PLoS Genet. 2021 Nov 22;17(11):e1009918. doi: 10.1371/journal.pgen.1009918 (PMC8648125; doi:10.1371/journal.pgen.1009918)
Supplement: S8 Fig — Number of distinct signals (i.e. number of significant PCs, see Methods) among differential genes (darker shade) and significant genes (lighter shade) detected by each method. Only genes tested in all three methods were considered in extracting differential genes exclusively found by one method but not the other two. The overall trend of CEWAS finding more distinct signals than MetaXcan and EpiXcan remains. (PDF) [file pgen.1009918.s014.pdf]

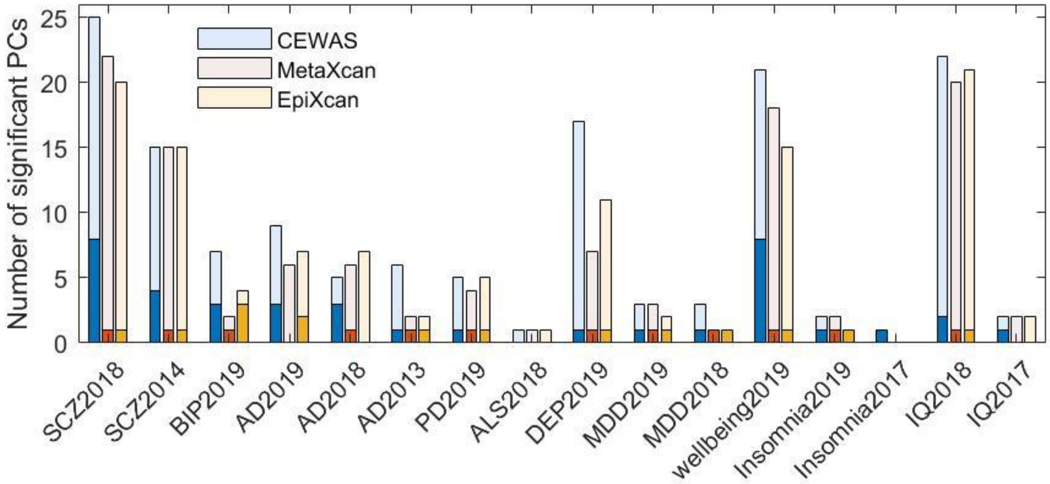

S8 Fig. Number of distinct signals among differential genes. Number of distinct signals (i.e. number of significant PCs, see Methods) among differential genes (darker shade) and significant genes (lighter shade) detected by each method. Only genes tested in all three methods were considered in extracting differential genes exclusively found by one method but not the other two. The overall trend of CEWAS finding more distinct signals than MetaXcan and EpiXcan remains.
