# Supplementary material for: Cascading epigenomic analysis for identifying disease genes from the regulatory landscape of GWAS variants
Source: PLoS Genet. 2021 Nov 22;17(11):e1009918. doi: 10.1371/journal.pgen.1009918 (PMC8648125; doi:10.1371/journal.pgen.1009918)
Supplement: S9 Fig — (a) The distance between TSS of differential genes exclusively found by CEWAS and their closest GWAS hits summarized. Each bar corresponds to the percentage of differential genes lying in a range of distance, e.g. a bar between 100 and 1000 corresponds to the percentage of differential genes with distance between 100 and 1000 base pairs. More than half of the differential genes are >100Kb away from any GWAS hits. (b) The percentage of differential genes exclusively found by CEWAS but missed by spatially mapping GWAS SNPs to their closest genes shown. Note that no differential genes were found for ALS2018, hence why the percentage is zero. (PDF) [file pgen.1009918.s015.pdf]

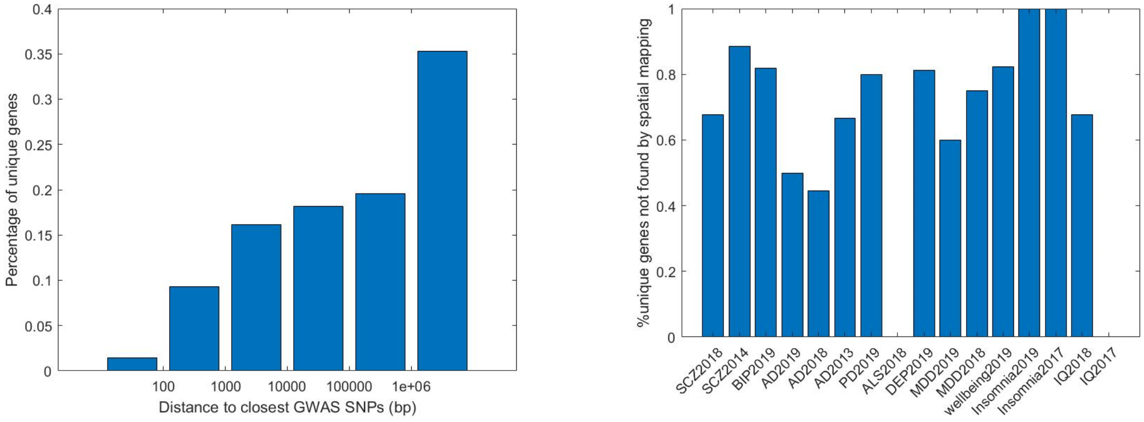

(a) (b)

S9 Fig. Comparison with spatially mapping GWAS SNPs. (a) The distance between TSS of differential genes exclusively found by CEWAS and their closest GWAS hits summarized. Each bar corresponds to the percentage of differential genes lying in a range of distance, e.g. a bar between 100 and 1000 corresponds to the percentage of differential genes with distance between 100 and 1000 base pairs. More than half of the differential genes are >100Kb away from any GWAS hits. (b) The percentage of differential genes exclusively found by CEWAS but missed by spatially mapping GWAS SNPs to their closest genes shown. Note that no differential genes were found for ALS2018, hence why the percentage is zero.
