# Supplementary material for: Cascading epigenomic analysis for identifying disease genes from the regulatory landscape of GWAS variants
Source: PLoS Genet. 2021 Nov 22;17(11):e1009918. doi: 10.1371/journal.pgen.1009918 (PMC8648125; doi:10.1371/journal.pgen.1009918)
Supplement: S10 Fig — The average correlation over GWAS for each gene set category displayed. The observed correlation suggests moderate similarity in enriched gene sets between CEWAS and the contrasted methods, which we confirmed by manual inspection. (PDF) [file pgen.1009918.s016.pdf]

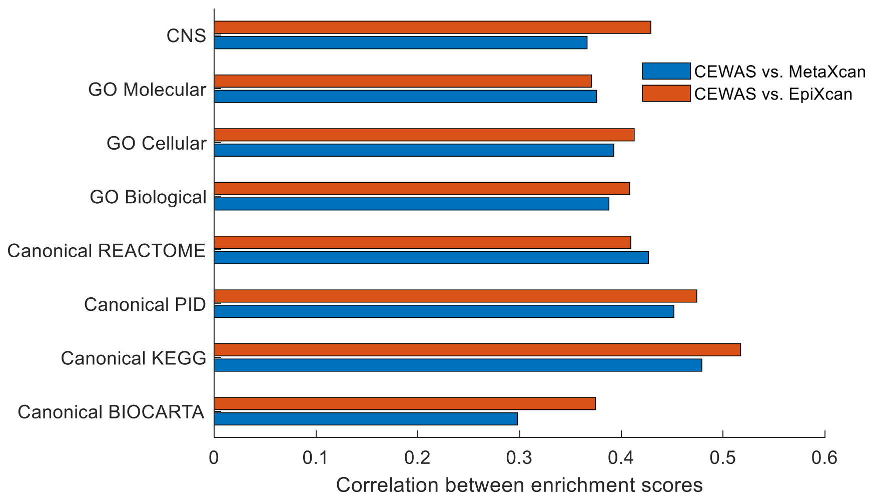

S10 Fig. Correlation of gene set enrichment scores between CEWAS and contrasted methods. The average correlation over GWAS for each gene set category displayed. The observed correlation suggests moderate similarity in enriched gene sets between CEWAS and the contrasted methods, which we confirmed by manual inspection.
