# Supplementary material for: Cascading epigenomic analysis for identifying disease genes from the regulatory landscape of GWAS variants
Source: PLoS Genet. 2021 Nov 22;17(11):e1009918. doi: 10.1371/journal.pgen.1009918 (PMC8648125; doi:10.1371/journal.pgen.1009918)
Supplement: S11 Fig — (a) Number of SNPs selected per CpG by elastic net. (b) Number of SNP selected per gene by elastic net. CEWAS tends to have more SNPs selected since 543 samples are available per CpG for selecting relevant SNPs with elastic net and each gene is associated with 9.62 CpGs on average, whereas MetaXcan and EpiXcan have only 534 samples per gene for SNP selection. (PDF) [file pgen.1009918.s017.pdf]

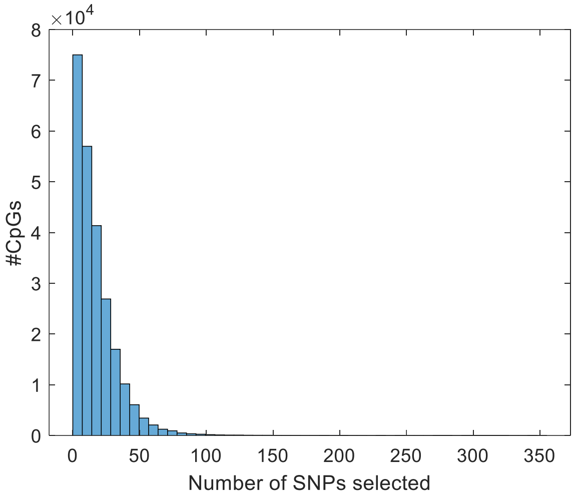

(a) Number of SNPs selected per CpG

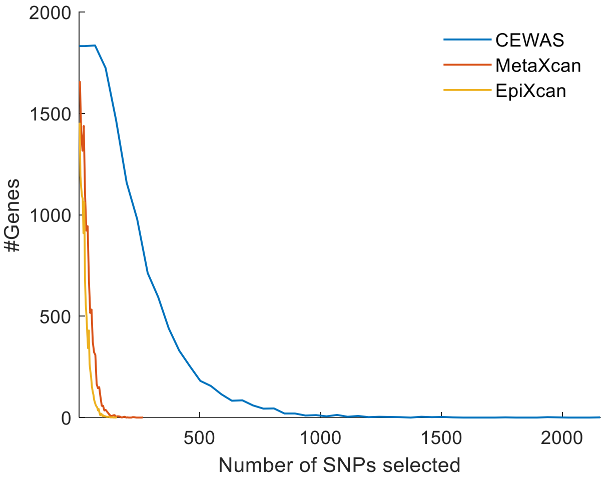

(b) Number of SNPs selected per gene.

S11 Fig. Number of selected SNPs. (a) Number of SNPs selected per CpG by elastic net. (b) Number of SNP selected per gene by elastic net. CEWAS tends to have more SNPs selected since 543 samples are available per CpG for selecting relevant SNPs with elastic net and each gene is associated with 9.62 CpGs on average, whereas MetaXcan and EpiXcan have only 534 samples per gene for SNP selection.
